# De-escalation as part of a global strategy of empiric antibiotherapy management. A retrospective study in a medico-surgical intensive care unit

**DOI:** 10.1186/cc9373

**Published:** 2010-12-17

**Authors:** Jérôme Morel, Julie Casoetto, Richard Jospé, Gérald Aubert, Raphael Terrana, Alain Dumont, Serge Molliex, Christian Auboyer

**Affiliations:** 1 Department of Anaesthesiology and Intensive Care Medicine, Centre Hospitalier Universitaire, Avenue A Raymond, Saint Etienne, 42055, France; 2 Department of Microbiology, Centre Hospitalier Universitaire, Avenue A Raymond, Saint Etienne, 42055, France

## Abstract

**Introduction:**

Most data on de-escalation of empirical antimicrobial therapy has focused on ventilator-associated pneumonia. In this retrospective monocentric study, we evaluated de-escalation as part of a global strategy of empiric antibiotherapy management irrespective of the location and the severity of the infection. The goal of this trial was to assess the application of a de-escalation strategy and the impact in terms of re-escalation, recurrent infection and to identify variables associated with de-escalation.

**Methods:**

All consecutive patients treated with empiric antibiotic therapy and hospitalized in the intensive care unit for at least 72 hours within a period of 16 months were included. We compared the characteristics and outcome of patients who have experienced de-escalation therapy with those who have not.

**Results:**

A total of 116 patients were studied corresponding to 133 infections. Antibiotic therapy was de-escalated in 60 cases (45%). De-escalation, primarily accomplished by a reduction in the number of antibiotics used, was observed in 52% of severe sepsis or septic shock patients. Adequate empiric antibiotic and use of aminoglycoside were independently linked with de-escalation. De-escalation therapy was associated with a significant reduction of recurrent infection (19% vs 5% *P *= 0.01). Mortality was not changed by de-escalation.

**Conclusions:**

As part of a global management of empiric antibiotherapy in an intensive care unit, de-escalation might be safe and feasible in a large proportion of patients.

## Introduction

The emergence of multidrug-resistant (MDR) pathogens is a major public health challenge and is directly correlated with over administration of antibiotics [1]. Controlling their use is thus a major objective of health. Responsible for more than one third of hospital admissions, infectious diseases are common in intensive care units [2]. Septic shock is present in 10% of intensive care unit (ICU) patients with a mortality rate of nearly 60% [3]. Early and adequate introduction of antibiotics improve survival in severe sepsis and septic shock patients [4-7]. Therefore, therapy such as broad-spectrum antibiotics and/or a combination of antibiotics must be started empirically. Guidelines recommend that physicians first combine broad-spectrum antibiotics followed by a reappraisal of the therapy as soon as bacteriological data and susceptibility tests are available in order to eventually reduce the number and the spectrum of the antibiotics [8,9].

This therapeutic strategy called de-escalation is particularly pertinent in case of serious infection [10-18]. Its feasibility is quite variable across centers with figures varying from 10% to 90% of cases [18,19]. The overwhelming majority of these studies were restricted to patients with ventilator associated pneumonia (VAP) [11-17]. However, empiric broad spectrum antibiotics are indicated in many others situations frequently encountered in an ICU [20,21].

We retrospectively evaluated the practice of de-escalation occurring over consecutive infections during a period of 16 months in a 10-bed intensive care unit, irrespective of type and severity of infection. The goal of the study was to assess the application of a de-escalation strategy on empirical antibiotics management. We particularly analyzed the clinical impact of this attitude in terms of re-escalation, recurrent infection and mortality and identified bio-clinical variables associated with de-escalation.

## Materials and methods

### Study design and patients

This retrospective observational study was conducted from January 2007 to April 2008 in a French teaching hospital. All consecutive patients admitted to the ICU (10 beds) and treated with empiric antibiotherapy have been included, irrespective of the origin and the severity of the suspected infection. Patients discharged from the ICU within 72 hours, patients with bone marrow aplasia, and patients admitted to the ICU already under antibiotherapy for more than 48 hours were excluded from the analysis. All the data have been reviewed and analyzed by three physicians involved in daily patient care. The study has been approved by the ethics committee of Saint Etienne University Teaching Hospital (number 20-2010) and informed consent was not required.

Antibiotic prescription was not protocolised in our unit. Empiric antibiotherapy was based on patients' characteristics, and the severity and location of the infection. The choice was made by the physician in charge of the patient according to our local ecology and pattern of resistance. After microbiological samples, broad spectrum antibiotics are usually prescribed in combination. Microbiologists are interviewed every morning to reassess this initial strategy. This reappraisal takes into account microbiological results, antibiotic susceptibility and also the clinical evolution of the patients. Every antibiotic change is systematically discussed with the staff at least three times a week. Tracheal secretions and urinary samples are collected twice a week for bacteriologic culture. In parallel, a specific search for MDR bacteria carriage is performed (nasal, throat, and rectum) at the admission and thereafter weekly.

### Definitions

De-escalation therapy was defined as either a switch to a narrower spectrum agent or the reduction in the number of antibiotics or the early arrest of antibiotic treatment.

A switch to a narrower antibiotic spectrum was considered when an antibiotic with activity against non-fermenting Gram-negative bacilli (nfGNB) (imipenem-cilastatin, piperacillin-tazobactam, ceftazidime or ciprofloxacin) was replaced by a molecule without nfGNB activity, an antibiotic with activity against meticillin resistant staphylococcus (MRS) was replaced by a molecule with an activity against methicillin sensible staphylococcus (MSS), or a third generation cephalosporin was replaced by a group A penicillin.

Reduction in the number of antibiotics was defined by the arrest of at least one antibiotic occurring before the fifth day of antibiotherapy.

Early arrest of antibiotics is defined as the early cessation of antibiotherapy (before the third day of treatment) either due to the absence of proven bacterial infection or due to the withholding of medical therapies.

Severe sepsis and septic shock were defined according to the classical criteria [22]. MDR bacteria were defined as methicillin-resistant *Staphylococcus aureus *and coagulase-negative staphylococci; Enterobacteria producing an extended-spectrum beta-lactamase or producing a cephalosporinase; and nfGNB resistant to piperacillin-tazobactam, ceftazidime, or imipenem-cilastatin or producing an extended-spectrum beta-lactamase (*Pseudomonas aeruginosa *and *Acinetobacter baumanii)*.

### Data collections

On admission the following variables were recorded: age, gender, Simplified Physiologic Score II (SAPS II), type of admission, MDR organisms. We also gathered information on the length of stay and on ICU mortality. Nosocomial infections were considered when they occurred after at least 48 hours of hospitalization. Immuno-compromised patients were defined as patients with an evolutive neoplasia or patients treated by immunosuppressive agents (corticoids for more than three months whatever the dose or chemotherapy). The diagnosis of ventilator associated pneumonia was established according to the French guidelines [23]: new infiltrates on chest radiograph, and at least one of the following criteria: body temperature >38°C, white blood cell count of <4,000/mm^3 ^or >12,000/mm^3^, and at least one of the following criteria: new onset of bronchial purulent sputum, alteration of arterial oxygenation, evocative pulmonary auscultation. Microbiological documentation is strongly recommended in cases with the presence of at least one microorganism at the concentration ≥10^4 ^Colony Forming Units/ml (CFU/ml) in the broncho-alveolar lavage sample or ≥10^5 ^CFU/ml in the tracheal secretions sample. The choice of empiric antibiotherapy may be helped by the result of the last systematic bacteriological samples. Urinary tract infection is difficult to diagnose in anesthetized patients. The definition used was the presence of at least one microorganism at the concentration ≥10^5 ^CFU/ml with symptoms and/or urinary catheter [23,24].

Empiric antibiotic treatment was deemed effective if at least one antibiotic molecule was active against bacteria responsible for the infection.

We defined re-escalation as the resumption of a broad spectrum treatment justified by a clinical worsening, not necessarily related to the initial infection, and a recurrent infection as the reappearance of an infection after the cessation of all antibiotic therapy.

### Statistical analysis

Qualitative variables were compared with chi-square test or Fisher exact test. Quantitative variables were compared with Student *t*-test. Univariate regression analysis was used to assess factors associated with de-escalation. All variables with a *P-*value < 0.1 determined by univariate regression model were entered into a multivariate logistic regression model. A *P-*value < 0.05 was considered statistically significant. Statistical analysis was performed using SAS version 9 (SAS Institute Inc., Cary, NC, USA).

## Results

Out of the 363 patients that have been hospitalized in our ICU over the 16-month study period, 116 met the criteria of inclusion, corresponding to 133 empiric antibiotic regimens. Because of infection recurrence, 15 patients received 2 antibiotic regimens and 1 patient, 3 antibiotics regimens.

De-escalation of empiric antibiotherapy was accomplished in 60 cases (45%), with a mean delay of 3.5 ± 0.7 days after their introduction. A decrease in the number of antibiotics was found in 19 cases (32%), a reduction of the spectrum in 5 cases (8%), and both approaches were found in 21 cases (35%). Antibiotic therapy was arrested early in 15 cases (25%): 6 pulmonary edema, 2 non-infectious interstitial pneumonia, 1 mycotic infection, 2 unknown etiologies, and 4 withholding medical therapies.

We analyzed two sub-groups of patients as a function of their de-escalation status: Group D corresponding to 60 empiric antibiotic regimens with de-escalation and Group ND corresponding to 73 empiric antibiotic regimens with no de-escalation.

Patients' admission characteristics are summarized in Table 1. No significant difference was noted between the two groups except a higher proportion of MDR bacteria carriage and less frequent primary diagnosis of infection at the admission for the patients of Group ND. Delay of empiric antibiotic introduction was not different between the two groups (5 ± 12 days and 5 ± 10 days for Group D and Group ND, respectively). Severity and type of infection were similar between the two groups except for mediastinitis (Table 2).

**Table 1:** Patients' characteristics at admission to intensive care unit. Comparison between groups D and ND

		Group D	Group ND	*P-*value
		*N *= 60	*n *= 73	
Age, years mean ± SD		62 ± 13	60 ± 17	0.46
SAPS II, mean ± SD		41 ± 15	40 ± 16	0.68
Immuno-compromised patients, n (%)		17 (28.3)	15 (20.5)	0.29
MDR, n (%)		1 (1.6)	7 (9.6)	0.05
Admission for infectious diseases		27 (45)	16 (22)	0.004
	Surgery	37 (61.6)	43 (59)	
Type of admission, n (%)	Medicine	21 (35)	20 (27.4)	0.10
	Trauma	2 (3.3)	10 (13.6)	
Length of stay, days mean ± SD		28 ± 33	24 ± 23	0.38

MDR, multidrug resistant pathogens; SAPS II: Simplified Acute Physiology Score.

**Table 2:** Characteristics of patients and type of infection at the moment of empiric antibiotics prescription.

		Group D *n *= 60	Group ND *n *= 73	*P-*value
Procalcitonine, mean ± SD, μg/l		7.8 ± 15	8.3 ± 18.5	0.87
Leukocyte count, mean ± SD, mg/l		13.6 ± 7.3	12.4 ± 6.1	0.34
Nosocomial infection, n (%)		54 (90)	62 (85)	0.38
	Sepsis	21 (35)	35 (48)	
Severity of infection, n (%)	Severe sepsis	23 (38.3)	24 (32.8)	0.3
	Septic shock.	16 (26.6)	14 (19.2)	
Type of infections, n (%)				
Ventilator-associated pneumonia		28 (46.6)	34 (46.5)	0.9
Pneumonia		13 (21.6)	24 (32.8)	0.15
Urinary tract infection		2 (3.3)	3 (4.1)	0.81
Catheter-related bacteriemia		1 (1.6)	0	0.28
Endocarditis		1 (1.6)	0	0.28
Mediastinitis		5 (8.3)	0	0.01
Peritonitis		6 (10)	7 (9.6)	0.97
Meningitis		1 (1.6)	0	0.28
Otorhinolaryngeal infection		0	1 (1.4)	0.34
Undetermined infection location		4 (6.6)	4 (5.5)	0.84

De-escalation occurred in 20 non-documented infections (15 early withdrawal and 5 reductions in the number of antibiotics). Microbiological details of documented infections are given in Table 3 and site by site in Table 4. The rate of antibiotic appropriateness was 43% for pulmonary infection (ventilator associated pneumonia and pneumonia), 80% for urinary tract infection, and 100% for the others sites. MDR bacteria and nfGNB were equally distributed between the two groups. An inadequate empiric broad-spectrum antibiotic therapy was more frequent in Group ND (27.5% versus 7.7% *P *= 0.02) and involved a MDR bacteria in 50% of cases. More details on antibiotics used can be found in Additional file 1. De-escalation was directly influenced by the number of empiric antibiotics used (Table 5). Only MRS-active antibiotics and aminoglycoside were associated with a more frequent de-escalation (Table 5). De-escalation therapy did not modify the duration of antibiotic therapy, 9.5 ± 6 days versus 10 ± 5 days for Group D and Group ND, respectively.

**Table 3:** Microbiologic characteristics of infectious episodes. Comparison between groups D and ND

	Group D *n *= 60	Group ND *n *= 73	*P-*value
Microbiological samples, n (%)	59 (98)	63 (86)	0.17
Positive microbiological documentation, n (%)	40 (66.6)	39 (53.4)	0.23
Inadequate empiric antibiotherapy, n (%) §	3 (7.7%)	11 (27.5%)	0.02
Bacteria related to infection, n (%):			
*Staphylococcus aureus*	11 (18.3) *	7 (9.6) *	0.18
CoNS	5 (8.3) $	0	0.02
*Streptococci *species	3 (5)	8 (11)	0.47
*Enterococci *species	2 (3.3)	2 (2.7)	0.89
Gram negative cocci	2 (3.3)	0	0.14
Enterobacteria	16 (26.6)	23 (31.5)	0.54
nfGNB	4 (6.6)	5 (6.8)	0.9
Others gram negative bacilli	7 (11.6)	5 (6.8)	0.14
Intracellular bacteria	0	1 (1.3)	0.34
MDR responsible for the infection, n (%)	6 (10)	7 (9.6)	0.97
Polymicrobial infections, n (%)	13 (21.6)	13 (17.8)	0.81
Infection recurrence, n (%)	3 (5)	14 (19)	0.01
MDR during the ICU stay, n (%)	6 (10)	14 (19.1)	0.1

§ Among documented infection; *1 methicillin resistant; $ 2 methicillin resistant.

CoNS, coagulase negative staphylococcus; MDR, multidrug resistant pathogen; nfGNB, non fermenting Gram-negative bacilli

**Table 4:** Site of infection among documented infections. Comparison between groups D and ND

Site of infection	Group D *n *= 40	Group ND *n *= 39	*P-*value
Ventilator-associated pneumonia, n (%)	19 (47.5)	23 (59)	0.07
Pneumonia, n (%)	11 (27.5)	12 (31)	0.25
Urinary tract infection, n (%)	2 (5)	3 (7.7)	0.32
Catheter-related bacteriemia, n (%)	1 (2.5)	0	
Endocarditis, n (%)	1 (2.5)	0	
Mediastinitis, n (%)	3 (7.5)	0	
Peritonitis, n (%)	3 (7.5)	1 (2.6)	0.4
Meningitis, n (%)	0	0	
Otorhinolaryngeal infection, n (%)	0	0	

**Table 5:** Empirical antibiotic treatment. Comparison between groups D and ND

	Group D *n *= 60	Group ND *n *= 73	P-value
Antibiotic with activity against MSS, n (%)	2 (3.3)	0	0.12
Antibiotic with activity against MRS, n (%)	21 (35)	12 (16.4)	0.01
ß-lactam antibiotic with no activity against nfGNB, n (%)	36 (60)	50 (68.5)	0.31
ß-lactam antibiotic with activity against nfGNB, n (%)	26 (43.3)	27 (37)	0.41
Quinolone (except ciprofloxacin), n (%)	5 (8.3)	8 (11)	0.53
Aminoglycoside, n (%)	20 (32.3)	3 (4.1)	<0.0001
Monotherapy, n (%)	14 (23.3)	44 (60.3)	<0.001
More than two antibiotics, n (%)	19 (31.7)	4 (5.4)	0.002

MRS, methicillin-resistant staphylococcus; MSS, methicillin-sensible staphylococcus; nfGNB, non fermenting Gram-negative bacilli.

A re-escalation of antibiotics occurred in four patients, on average 3.75 ± 1.5 days after de-escalation and was due in half of the cases to MDR *P. aeruginosa *strain. Recurrent infections were more common in Group ND (19% versus 5%, *P *= 0.01), with 50% caused by MDR bacteria (Table 3). Mortality was not different between the two groups 18.3% vs 24.6% for Group D and Group ND, respectively. In multivariate analysis, only aminoglycosides and adequate antibiotic therapy were independent factors associated with de-escalation (Table 6). MDR pathogens at admission and monotherapy were found not to be associated with de-escalation (Table 6).

**Table 6:** Multivariate logistic regression analysis to assess factors associated with de-escalation therapy

	OR (95% IC)	*P-*value
MDR at admission	0.02 (0.00; 0.36)	0.008
Aminoglycoside	18.08 (2.25; 145)	0.006
Monotherapy	0.28 (0.12; 0.63)	0.002
Adequate antibiotic therapy	5.25(1; 27.4)	0.049

MDR, multidrug resistant pathogen

## Discussion

In this retrospective study, de-escalation, as a global management of antibiotherapy in the ICU, occurred in 45% of the cases. De-escalation was possible irrespective of the severity of the infection, and more frequently translated into a reduction of the number of antibiotics rather than a reduction of the spectrum. Although the study was not powered for clinical outcomes, de-escalation seems to be safe with no excess of mortality and might even allow a reduction in recurrent infections.

Many variables play a role in de-escalation and may explain the large variation of incidence found in the literature; 6.1% [19] to 98% [18].

First of all there is no consensual definition for de-escalation. De-escalation therapy was defined as either a switch to a narrower spectrum agent, or the reduction in the number of antibiotics, or the early arrest of antibiotic treatment [10,11,16,17]. By focusing on two factors known to facilitate MDR emergence, namely the broad spectrum antibiotics and the number of antibiotics associated, this definition is probably the most relevant from a microbiological standpoint [25,26].

An overwhelming majority of the studies published on de-escalation so far has focused on VAP [10-16]. VAP is traditionally the main reason for antibiotic administration in the ICU, and as such, represents in our study a substantial proportion of infections. Nearly 40% of empiric antibiotherapies are administered for an infection located other than in the respiratory tract [27], illustrating our objective to assess de-escalation as part of a global antibiotherapy management for non-selected infections. In this context, we show that de-escalation is feasible in many other situations such as mediastinitis or peritonitis, situations which like VAP also require broad spectrum antibiotics.

We confirm that de-escalation is achieved more frequently by reducing the number of drugs rather than by reducing the spectrum of antibiotic therapy [10,15,16]. Monotherapy is accordingly independently associated with the absence of de-escalation. Aminoglycosides were the antibiotics most frequently de-escalated. The risk of nephrotoxicity and the necessity to adapt their posology are probably one explanation.

Absence of positive microbial documentation did not apparently influence our strategy of de-escalation. Of note, 70% of cases without microbial documentation were obviously non-bacterial disease and thus the decision to de-escalate was easy. De-escalation is, however, more problematic when the clinician has a strong suspicion of bacterial infection with no positive microbial documentation [11,15]. This concept of de-escalation in patients with no microbial documentation is not widely accepted and is still a matter of discussion. Early clinical evolution under antibiotics may help the clinician with this choice [28]. In the case of documented infection, there is no consensus as to whether de-escalation should extend to infections with MDR pathogens. Although de-escalation seems to be possible when such pathogens are directly responsible for the infection [10,16], this strategy remains restricted to non-MDR pathogen-induced infections [11,13]. In two successive works Rello *et al. *showed an increase in de-escalation rate (6.1% vs 31.4%), while the incidence of *P. aeruginosa *decreased from 50% to 15% [11,19]. De-escalation was only done in 2.7% of infections with MDR pathogens compared with 49.3% in those with other agents [11]. Whether the decrease of *P. aeruginosa *incidence is the cause or the consequence of the increase in de-escalation strategy is not clear. On the other hand, Leone *et al. *reported a de-escalation rate of 54% for VAP due to *P. aeruginosa*, *A. baumanii *and methicillin resistant *S. aureus *as compared to 39% for VAP due to other bacteria [10]. In this study incidence of MDR agents was nearly 16%. With less than 10% of MDR pathogen incidence, we are not powered to analyze the influence of MDR pathogen identification on our strategy of de-escalation.

Consequences of de-escalation therapy on the emergence of bacterial resistance are difficult to analyze. We did not find a lower incidence of MDR acquisition in Group D (10% vs 19.1%, *P *= 0.10).

In our study, severity of the infection did not impact our decision to de-escalate. Among the patients with severe sepsis or in septic shock (near 60% of our cohort), de-escalation was possible in 65% of the cases which is in agreement with what has been previously reported [17].

Interestingly, recurrent infections were increased in Group ND (19% versus 5%, *P *= 0.01). Singh *et al. *compared a de-escalation strategy (short course of empiric antibiotics therapy) to standard care. Antimicrobial resistance and/or superinfections were documented in 15% of the patients in the experimental group and in 35% of the patients in the control group [28]. A decrease in mortality rate and length of stay had sometimes been described with de-escalation [10,13,15,16]. The number of patients was not large enough to detect an impact of de-escalation on this outcome in this study. In our study, four re-escalations (6.6%) occurred, which is comparable of Leone's study (6%) [10]. We did not record a decrease in antibiotic duration in Group D.

The main limit of this study is its retrospective design. We aimed to get a comprehensive picture of our daily practice. While a prospective gathering of data would have probably influenced our attitude in favour of de-escalation, it would be the clinical trial design of choice to answer the question of de-escalation efficiency. Moreover, delay in de-escalation might be considered long (3.5 ± 0.7 days) with respect to the current guidelines (two to three days) [8], but maybe not in respect to clinical practice [10]. The study is not powered to detect an impact of de-escalation on MDR emergence, although this is one of the main aims of this strategy. The implementation of a de-escalation directed protocol for antibiotic management compared to a more liberal strategy with no de-escalation may answer this question.

## Conclusions

As part of a global management of empiric antibiotherapy in an ICU, de-escalation might be safe and feasible in a large proportion of patients and infections. De-escalation is not realized in more than 50% of the antibiotherapy. Identification of the reasons that impair the decision towards de-escalation could eventually help to curb the clinician's reluctance to generalize this strategy.

## Key messages

• De-escalation is feasible in many infections other than ventilator associated pneumonia.

• De-escalation is mostly accomplished by a reduction in the number of antibiotics used.

• Adequate empiric antibiotic and use of aminoglycosides were independently linked with de-escalation.

## Abbreviations

CFU/ml: Colony Forming Units/ml; MDR: multi-drug resistant; MRS: methicillin-resitant staphylococcus; MSS: methicillin-sensible staphylococcus; nfGNB: nonfermenting Gram negative bacilli; SAPS II: Simplified Physiologic Score II; VAP: ventilator-associated pneumonia

## Competing interests

The authors declare that they have no competing interests.

## Authors' contributions

JM, JC and CA participated in the design of the study. GA carried out microbiological analysis. JM and SM performed the statistical analysis. JC, RJ and CA gathered and analyzed the data. JM, JC, SM and CA drafted the manuscript. All authors read and approved the final manuscript.

## Supplementary Material

Additional file 1 **Supplementary material**. Description of empirical antibiotics used and description of empirical antibiotics association among documented infections. Click here for file
