# Supplementary material for: De-escalation as part of a global strategy of empiric antibiotherapy management. A retrospective study in a medico-surgical intensive care unit
Source: Crit Care. 2010 Dec 17;14(6):R225. doi: 10.1186/cc9373 (PMC3219998; doi:10.1186/cc9373)
Supplement: Additional file 1 — Supplementary material. Description of empirical antibiotics used and description of empirical antibiotics association among documented infections. [file cc9373-S1.DOC]

Description of empirical antibiotics used

|  | Group D (n=60) | Group ND (n=73) |
| --- | --- | --- |
| Antibiotics with activity against MSS, n (%) | 2 (3.3) | 0 |
| cloxacillin | 2 (3,3) | 0 |
| Antibiotics with activity against MRS, n (%) | 21 (35) | 12 (16.4) |
| glycopeptide | 18 (30) | 6 (8,2) |
| linezolid | 3 (5) | 7 (9,6) |
| rifamycin | 3 (5) | 0 |
| ß-lactam antibiotics with no activity against nfGNB, n (%) | 36 (60) | 50 (68.5) |
| Amoxicillin/ clavulanate | 19 (31,6) | 31 (42,4) |
| Third generation cephalosporin | 16 (26,6) | 18 (24,6) |
| Amoxicillin | 1 (1,6) | 1 (1,3) |
| Antibiotics with activity against nfGNB, n (%) | 26 (43.3) | 27 (37) |
| Carbapenem | 2 (3,3) | 3 (4,1) |
| Piperacillin/tazobactam | 19 (31,6) | 17 (23,3) |
| Ceftazidime | 2 (3,3) | 1 (1,3) |
| Ciprofloxacin | 8 (13,3) | 9 (12,3) |
| Quinolone (except ciprofloxacin), n (%) | 5 (8.3) | 8 (11) |
| Aminoglycoside, n (%) | 20 (33.3) | 3 (4.1) |
| Amikacin | 15 (25%) | 3 (4,1) |
| Gentamicin | 5 (8.3%) | 0 |

MRS: methicillin-resistant staphylococcus; MSS: methicillin-sensible staphylococcus; GNB: Gram-negative bacilli; nfGNB: non fermenting Gram negative bacilli

Description of empirical antibiotic association among documented infections

|  | Group D (n=40) | Group ND (n=39) |
| --- | --- | --- |
| Anti MRS+anti GNB | 3 | 5 |
| Anti MRS+anti nfGNB | 3 | 6 |
| Anti nfGNB+quinolone | 7 | 14 |
| Anti nfGNB+aminoside | 6 | 3 |
| Anti GNB+quinolone | 1 | 3 |
| Anti GNB+aminoside | 2 |  |
| Anti MSS+aminoside | 1 |  |
| Anti MSS+aminoside+nfGNB | 1 |  |
| Anti MRS+aminoside+nfGNB | 5 |  |
| Anti GNB | 4 | 2 |
| Anti nf GNB | 4 | 3 |
| Anti MRS+anti GNB+Q | 3 | 3 |

MRS: Antibiotics with activity against methicillin-resistant staphylococcus; MSS: Antibiotics with activity against methicillin-sensible staphylococcus; GNB: ß-lactam antibiotics with no activity against nf Gram-negative bacilli; nfGNB: ß-lactam antibiotics with activity against nfGNB non fermenting Gram negative bacilli; Q: all Quinolone (ciprofloxacin include)
